# From muscles to motion: the role of sensor layout and physiological factors in hand motion decoding

**DOI:** 10.1038/s41598-026-59979-6

**Published:** 2026-07-01

**Authors:** Daniel Andreas, Anany Dwivedi, Claudio Castellini, Philipp Beckerle

**Affiliations:** 1 Chair of Autonomous Systems and Mechatronics, Friedrich-Alexander-Universität Erlangen-Nürnberg, 91054 Erlangen, Germany; 2 Artificial Intelligence (AI) Institute, Division of Health, Engineering, Computing and Science, University of Waikato, Hamilton, 3216 New Zealand; 3 Assistive Intelligent Robotics Lab, Friedrich-Alexander-Universität Erlangen-Nürnberg, 91054 Erlangen, Germany; 4 Department of Artificial Intelligence in Biomedical Engineering, Friedrich-Alexander-Universität Erlangen-Nürnberg, 91054 Erlangen, Germany

**Keywords:** Myoelectric decoding, Multimodal fusion, Hand kinematics, Surface electromyography, Force myography, Deep learning, Engineering, Health care, Neuroscience, Physiology

## Abstract

Despite substantial progress in decoding biosignals for human motion prediction, the influence of participant- and experiment-related factors on the decodability of these signals has received comparatively little attention. This study evaluates the continuous prediction of hand and wrist joint flexion using the MyoKi database, which comprises surface electromyography, inertial measurement units, and force myography data from 35 participants without disabilities performing 74 daily-life tasks. Unlike existing datasets, MyoKi includes tasks that mimic real-world scenarios by allowing natural movement variations and muscle fatigue. Using a long short-term memory neural network, the impact of participant- and experiment-related factors on decoding accuracy was investigated. Our results show that both expanding sensor coverage to additional muscle regions and combining multiple sensor modalities significantly improve decoding performance, with the greatest gains observed for joints controlled by extrinsic muscles. Muscle fatigue, recording time, and participant characteristics such as weight also influenced model accuracy. However, decoding of movements driven by intrinsic hand muscles remains challenging due to anatomical limitations. These findings highlight the importance of sensor placement and multimodal fusion for myoelectric decoding and provide guidance for optimizing sensor configurations in future prosthetic and robotic applications.

## Introduction

Despite advances in robotic autonomy, human-in-the-loop control of the human hand remains essential in safety-critical and high-precision applications such as teleoperation, surgical robotics, and prosthetics. In the United States alone, the population living with limb loss was estimated at approximately 1.6 million in 2005 and is projected to more than double to over 3.5 million by 2050, largely due to aging populations and vascular disease^1^. More recent reviews confirm this upward trend globally, reporting sustained increases in amputation incidence and prosthetic demand across both high- and low-income countries^2^. This growing demand for prosthetic and rehabilitation technologies underscores the need for reliable, intuitive human-machine interfaces that effectively translate hand intent into robotic action. Recent advances in artificial intelligence (AI) and machine learning (ML) have enabled increasingly sophisticated models to decode biosignals for the control of robotic and prosthetic hands^3–6^. Traditional ML approaches for myoelectric control have commonly relied on handcrafted feature extraction combined with regression or classification models such as support vector machines, random forests, or linear regression methods^7^. However, these approaches often struggle to fully capture the complex nonlinear and temporal relationships present in biosignals during continuous hand movements. Deep learning (DL), a subset of ML based on multi-layer neural networks, has become a dominant approach for biosignal decoding, with recurrent architectures such as long short-term memory (LSTM) networks^8^ being particularly well suited for sequential and time-dependent data. LSTM networks can model temporal dependencies across consecutive signal windows and have shown strong performance in continuous myoelectric regression tasks due to their ability to retain relevant temporal information^8^. Besides LSTMs, other commonly used architectures for continuous myoelectric decoding include temporal convolutional networks (TCNs)^9^, CNN-LSTM hybrids^10^, gated recurrent units (GRUs)^7^, and transformer-based models^11^, which similarly aim to capture temporal structure in EMG signals. Many previous studies focused on improving decoding performance and developing increasingly sophisticated model architectures. What appears to be more relevant, however, is to understand the underlying causes of the low robustness of decoding models, especially under complex daily-life situations. This requires the careful investigation of participant- and experiment-related factors on hand motion decoding performance. Addressing these factors is particularly important because decoding models intended for robotic and especially prosthetic applications must ultimately operate under highly variable real-world conditions rather than carefully controlled laboratory environments. Especially in prosthetic applications, insufficient robustness to such variability could limit functionality and thus negatively impact embodiment of the prosthesis (i.e., the sense of ownership and agency over a prosthetic device, often linked to its functional integration in daily-life use)^12^.

Table 1 provides an overview of previous literature investigating participant- and experiment-related factors influencing hand motion decoding performance. The present study shall validate, build upon, and extend those findings by systematically evaluating decoding performance in the continuous prediction of hand and wrist joint flexion from biosignals recorded on participants’ right arms. The investigated variables are summarized in Table 2 and were grouped into participant- and experimental-related factors.

**Table 1:** Overview of previous work investigating participant- and experiment-related factors influencing hand motion decoding performance.

Factors	Study	Modalities	Main findings
Sensor modality combinations	Jiang et al.^13^, Choi et al.^14^, Chen et al.^15^	EMG + FMG	FMG provided complementary mechanical information, improving decoding performance. Combining both modalities leads to higher accuracy compared to each individual signal
	Krasoulis et al.^16^	EMG + IMU	Increase in gesture classification accuracy when combining modalities
	Gharibo et al.^17^	EMG + FMG + IMU	Increase in gesture classification accuracy for each added modality
Muscle region selection	Pelaez-Murciego et al.^18^	HD-EMG	Demonstrated that strategically selected forearm EMG channels maintain robust decoding performance with fewer electrodes
	Prakash et al.^19^	HD-EMG	Showed that decoding performance depends strongly on EMG sensor placement
Sensor shift and cross-subject generalization	Yang et al.^20^	EMG	Results indicate lower wrist motion decoding performance for sensor shift or cross-arm and cross-subject generalization
Muscle fatigue	Merletti et al.^21^, Viitasalo et al.^22^	EMG	Reported fatigue-related variations in EMG spectral characteristics
	Qing et al.^23^	EMG	Reported that muscle fatigue reduced gesture decoding performance by 7%
Subcutaneous fat	Oliveira et al.^24^, Cifrek et al.^25^	EMG	Increased muscle-electrode distance negatively affected EMG signal quality. Reported attenuation and spatial smoothing of EMG signals caused by adipose tissue
Arm position variability	Qing et al.^23^	EMG	Reported a decrease in gesture classification accuracy for variations in arm orientation between training and testing data
Sex	Freitas et al.^26^	EMG	No significant difference among female and male participants regarding classification accuracy
Age, skin hydration, skin elasticity, BMI	Gowda et al.^27^	EMG	Suggest no relevant impact of age, skin hydration, skin elasticity, and BMI on decoding accuracy

Although previous studies have investigated individual factors influencing hand motion decoding performance, important limitations remain. Existing work on multimodal sensing has focused primarily on discrete gesture classification rather than continuous regression of hand kinematics^13–15,17^. Similarly, studies investigating spatial sensor distributions and HD-EMG electrode placement mainly analyzed EMG-only configurations under constrained laboratory conditions^18,19^. Furthermore, physiological and demographic factors such as muscle fatigue, subcutaneous tissue, age, and sex have rarely been evaluated jointly within realistic multimodal decoding scenarios involving variable arm orientations and daily-life tasks. Consequently, it remains unclear which participant- and experiment-related factors most strongly influence robust continuous hand motion decoding in ecologically valid settings.

Regarding participant-related factors, we investigate whether variables such as age, sex, and subcutaneous tissue are associated with decoding performance, as previous studies have already shown the negative impact of subcutaneous tissue on EMG signal quality^24,25,28^. Oliveira et al.^24^, for instance, showed that increased muscle-electrode distance, associated with greater subcutaneous fat thickness, negatively affects EMG signal quality. The authors suggested that the increased distance between muscles and surface electrodes leads to similar, more uniform muscle activation signals, and with that effectively decreases the resolution of the acquired signal. Further, we investigate the impact of experiment-related factors, including various combinations of sensor modalities, namely EMG, FMG, accelerometer (ACC), and gyroscopic (GYR) data, on decoding performance to identify suitable setups for future applications. Several previous studies have demonstrated the benefit of multimodal sensing for myoelectric control^13–15,29–32^. Jiang et al. combined EMG and FMG in a co-located design for gesture classification and reported improved robustness compared to each individual signal^13^. Similarly, Choi et al. and Chen et al. demonstrated that FMG provides complementary mechanical information to EMG, which can improve decoding stability in multimodal hand-motion recognition systems^14,15^. However, these studies focused primarily on discrete gesture classification tasks rather than continuous regression of hand kinematics. We now aim to validate these findings for regression approaches.

**Table 2:** Overview of investigated factor categories and corresponding variables.

Category	Investigated variables
Participant-related factors	Age, sex, weight, height, forearm circumference, subcutaneous tissue (skinfold thickness at triceps), handedness, muscle fatigue
Experiment-related factors	Recording time, sensor modality combinations, muscle region selection

Performing such analyses requires large amounts of muscular activity data alongside hand kinematics acquired under realistic daily-life conditions. Previous datasets have provided valuable resources for hand motion decoding, but exhibit specific limitations. For instance, several studies focused on single-plane hand or finger movements under highly controlled conditions, with participants performing repetitive gestures from a fixed posture^16,33–39^, such as in the work of Krasoulis et al., where participants performed repetitive hand and wrist movements while maintaining a fixed seated posture and arm position^16^. Other datasets lacked multimodal signal acquisition and purely relied on sEMG^38–41^. Other works lacked the coverage of daily-life variability, such as different arm orientations^37–41^ or object interactions^41–43^, with Hu et al.^43^ focusing on 12 distinct hand gestures not including any objects. Additionally, many datasets included small or demographically homogeneous participant cohorts, as in Furmanek et al.^42^, which mostly contained data from young males, limiting analyses of inter-individual differences.

By contrast, MyoKi incorporates tasks with variable arm orientations, object positions, and natural whole-body movements, allowing variability in movement execution and the presence of muscle fatigue, both of which can impact decoding performance^23^. The database also contains a roughly balanced distribution across age groups and sex, supporting studies of robustness and inter-individual variability under conditions that better approximate everyday scenarios. The MyoKi database^44^ contains data from 35 participants without disabilities and was designed to support research in continuous hand motion decoding. It is the first publicly available multimodal database of surface electromyography (sEMG), inertial measurement units (IMUs) acquiring accelerometer (ACC) and gyroscopic (GYR) data, and force myography (FMG) alongside hand kinematics^45^.

In prosthesis applications, for instance, sensor placement is often constrained by the limited available surface on the residual limb and by the geometry of the prosthetic shaft. Therefore, we additionally investigate the contribution of different muscle regions as an experimental factor influencing decoding performance, namely the lower/mid forearm, upper forearm, and upper arm. Although such analyses have been done in previous studies using high-density EMG (HD-EMG)^18,19,46,47^. Prakash et al.^19^, for instance, tested different sensor layouts and investigated the roles of the muscles targeted in the ideal sensor combination. However, those studies lacked multimodal sensing, did not consider the upper arm as a potential muscle region, and the performed tasks were often less complex.

In summary, the aim of this work is first, to quantify the impact of participant- and experiment-related factors on the decodability of muscle activity into hand kinematics, and second, to identify which input modalities and muscle regions contribute most to robust decoding. By addressing these questions in the context of a realistic, multimodal dataset within three evaluation cases, this study aims to provide insights into both the design of sensor layouts and the robustness of decoding models for future prosthetic and robotic hand control applications.

The remainder of this article is organized as follows: the "Methods" describe the MyoKi database used for the evaluation in addition to the preprocessing pipeline, the neural network architecture, and model training. The "Results" present the decoding performances and statistical analyses across the three evaluation cases. Finally, the "Discussion" highlights the implications of the findings, study limitations, and relevance for future prosthetic and robotic hand control systems.

## Methods

All data analyzed in this work were previously published in the MyoKi database^45^. The original study was approved by the Institutional Ethics Committee of the Friedrich-Alexander-Universität Erlangen-Nürnberg (24-439-S 2024-12-11), and all experimental protocols were carried out in accordance with relevant guidelines and regulations. Informed consent was obtained from all participants by the original investigators. The MyoKi database provides multimodal recordings of muscular activity and hand kinematics collected during 74 realistic daily-life tasks performed by 35 participants without upper-limb impairments.

The database comprises two participant subsets. In the first subset (P01 to P25), surface electromyography (sEMG) and inertial measurement unit (IMU) data were recorded, whereas in the second subset (P26 to P35), additional force myography (FMG) data were acquired using the multimodal bracelet by Andreas et al.^48^. In total, 12 sEMG channels and 9 IMUs (each including a 3-axis accelerometer and gyroscope) were distributed across the participants’ right arms. Six sEMG sensors (1–6) were equally spaced around the upper forearm using the multimodal bracelet to ensure reproducible placement, as shown in Fig. 8, with each sensor co-located with four FMG channels in subset 2. Two additional sEMG sensors were placed on the mid/lower forearm, and four sensors on the upper arm (biceps, triceps, and anterior/posterior deltoid). sEMG measures the electrical activity generated during muscle contractions using electrodes placed on the skin surface^49^. It provides direct information about neural muscle activation but is sensitive to electrode shifts, skin conditions, and electromagnetic noise^29,50^. Mechanomyography (MMG), commonly recorded using ACCs, captures mechanical muscle vibrations and soft-tissue oscillations produced during muscle contractions^51^. Compared to sEMG, MMG is less affected by electrical interference but can be sensitive to motion artifacts and external vibrations^52,53^. GYR signals measure angular velocity and provide information about forearm and wrist orientation during movement. Although they do not directly measure muscle activity, they provide global kinematic context that can improve decoding robustness across changing arm positions and dynamic tasks. FMG measures pressure and volumetric changes of the limb caused by muscle contractions^54^. FMG captures mechanical tissue deformation rather than electrical activation and provides stable sensing^54,55^. Hand kinematics were captured at 18 finger and wrist joints using the CyberGlove by CyberGlove Systems LLC, CA, USA.

**Fig. 1:**
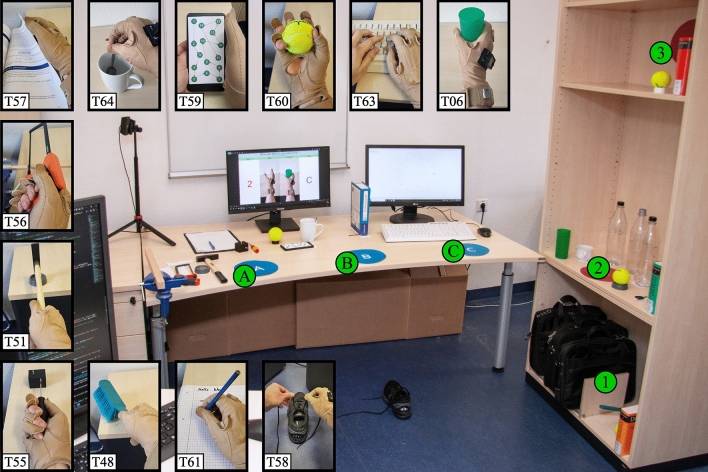
Experimental setup used for data acquisition. Markers 1, 2, and 3 indicate the initial object positions for the pick-and-place tasks with objects of different sizes and weights (such as T06), whereas A, B, and C denote the corresponding target positions. The task set further included isolated joint movements and everyday activities, such as tying a shoe (T58), hand writing (T61), using a hand broom (T48), screwing a screw (T55), hammering (T51), sawing (T56), turning pages (T57), stirring a cup of tea (T64), tapping on a smartphone (T59), twisting a ball (T60), or typing (T63).

The 74 tasks were designed to reflect the variability and complexity of daily activities and were systematically categorized across multiple dimensions (see Task_categorization.xlsx from the MyoKi database^44^). Tasks differed in vertical location (above, at, or below table height), movement distance (short, medium, or long) and direction (medial or lateral), grasp type (cylindrical, spherical, hook, tripod, pinch, lumbrical, or complex), and wrist involvement (supination/pronation, radial/ulnar deviation, flexion/extension). They also varied in required force, ranging from low to high depending on object weight, and in manual involvement, with tasks executed either unimanually or bimanually. Figure 1 provides an overview of the experimental setup along with examples of the performed tasks. The task set included both everyday activities, such as stirring a cup of tea (T64), turning pages (T57), typing (T63), or tapping on a smartphone (T59), and controlled pick-and-place actions with objects of different sizes and weights (such as T06). In addition, isolated joint movements were incorporated, covering finger flexion and extension, abduction and adduction, and wrist supination, pronation, deviation, and flexion/extension. Each task was repeated six times, with precise labels for task identity and repetition provided in the dataset. Each repetition corresponds to one complete execution of the full task protocol.

Data were sampled at modality-specific rates (EMG: 1998.96 Hz, IMU: 146.91 Hz, FMG: 89.44 Hz, hand kinematics: 80.85 Hz) and synchronized across modalities. Participant information (see Participant_information.xlsx from the MyoKi database^44^) includes all participant-related variables from Table 2 (except muscle fatigue) and are investigated regarding their impact on hand motion decoding performance. The cohort consisted of 35 individuals (17 female, 18 male) covering a broad age range (18–29: 11; 30–44: 8; 45–59: 9; 60+: 7). Participant-related measurements included weight ($76.6\pm 14.7$ kg), forearm circumference ($27.1\pm 2.7$ cm), height ($174.4\pm 8.6$ cm), and handedness (31 right, 4 left). This variability in characteristics provides an opportunity to examine how participant-related factors influence the decoding of biosignals into hand and wrist kinematics.

For the present work, several neural network architectures were tested for decoding multimodal biosignals from the participants’ right arms into continuous 18-channel hand and wrist kinematics, including an LSTM and GRU network^8^ with hand-crafted features derived from established literature, a CNN-LSTM model with two convolutional layers for automatic feature extraction, and a transformer-based model. Both the GRU and the CNN-LSTM performed slightly worse than the feature-based LSTM, while the transformer model required substantially longer training times, making it impractical for subject-specific training and exceeding the available GPU memory constraints. LSTM networks are an established tool for decoding EMG signals, known for their ability to model temporal dynamics in biosignals. Since our goal is not to achieve the highest possible decoding performance, but rather to study how participant- and experiment-related factors influence performance, we focus on variations in model performance across conditions rather than absolute performance levels. Thus, the LSTM architecture was employed for the remainder of the study using the same architecture as in the original MyoKi database descriptor^45^, since it provided the best balance between decoding accuracy and computational efficiency. The LSTM network architecture consisted of two recurrent layers with 4096 hidden units each, rectified linear unit (ReLU) activations, and a dropout probability of 0.1. Training was conducted for 200 epochs (with early stopping criteria based on validation loss and a patience of 30 epochs) using the Adam optimizer, configured with a learning rate of 5.72589$\times$10$^{-5}$, weight decay of 0.0001, and a batch size of 128. Mean squared error (MSE) was used as the loss function. Input signals were segmented into 250 ms windows with a 150 ms overlap, following prior EMG decoding studies reporting improved classification performance for window lengths in the range of approximately 250 ms^56^. Electromyographic recordings were band-pass filtered between 20–500 Hz with a 4th-order Butterworth filter and further processed with a 50 Hz notch filter to suppress power-line interference. All input channels and glove outputs were normalized to zero mean and unit variance prior to feature extraction. For each modality, a comprehensive set of time- and frequency-domain features, adopted from the MyoKi data descriptor^45^, was extracted to avoid unnecessarily constraining the representational capacity of the model and to capture a broad range of signal characteristics commonly used in myoelectric and multimodal motion decoding^57–59^:

- *Electromyography (EMG)* mean value (MV), variance (VAR), root mean square (RMS), signal range (SR), waveform length (WL), zero crossing (ZC), mean frequency (MNF), median frequency (MDF), spectral entropy (SE), skewness (Skew), kurtosis (Kurt), entropy, mean absolute value (MAV), integrated EMG (IEMG), slope sign change (SSC), log determinant (LogDet), difference absolute standard deviation value (DASDV), and average amplitude change (AAC).
- *Accelerometer (ACC)* MV, VAR, RMS, SR, WL, ZC, MNF, MDF, SE, Skew, Kurt, Entropy, MAV, IEMG, SSC.
- *Gyroscope (GYR)* MV, VAR, RMS, SR, WL, ZC, MNF, MDF, SE, Skew, Kurt, Entropy, MAV.
- *Force myography (FMG)* MV, VAR, RMS, SR, WL, ZC, MNF, MDF, SE, Skew, Kurt, Entropy, MAV, IEMG, SSC.

The extracted feature vectors were provided as inputs to the LSTM, which generated continuous predictions of finger flexion across all CyberGlove sensors. We used the non-calibrated glove data from the dataset. However, since neural networks are robust to linear scaling, this choice is not expected to affect decoding performance. To evaluate decoding performance, the coefficient of determination ($\textrm{R}^{2}$ score) was employed as the primary metric. The $\textrm{R}^{2}$ score was computed over the continuous regression output of all 18 hand and wrist kinematic channels jointly, aggregated across all time samples of the respective test repetition. The $\textrm{R}^{2}$ score quantifies how well the predicted joint angles match the ground-truth data from the CyberGlove and serves as a reliable measure of decoding accuracy. For robust comparisons, all evaluation cases employed a k-fold cross-validation strategy based on repetitions. Each participant in the MyoKi database performed six repetitions (i.e., six full recordings of the complete task set), which were used to construct six folds. In each iteration, one repetition served as the test set, while another randomly selected repetition was used for validation, and the remaining four repetitions were used for training, with data being shuffled before being fed into the model. This procedure was repeated until every repetition had served once as the test set, ensuring that results were not biased by a particular train-test split. For each participant, we computed an overall $\textrm{R}^{2}$ score (averaged across all 18 kinematic channels) as well as joint-wise $\textrm{R}^{2}$ scores.

Three evaluation cases were employed to investigate the impact of participant-related factors, sensor modalities, and muscle region on the decodability of muscular activity. The first evaluation case examined factors influencing decoding performance related to participant characteristics (such as age, body fat, sex), muscle fatigue, recording time, and joint-specific accuracy. Muscle fatigue was quantified through the median frequency (MDF) feature of the EMG signal. MDF was extracted by first applying a bandpass filter (20–500 Hz) and a notch filter (50 Hz) to the EMG signals. For each signal segment, the power spectral density was estimated using Welch’s method, and the MDF was computed as the frequency that divides the power spectrum into two regions with equal power. This was done per repetition and per participant. We then assessed correlations between this fatigue-related feature and decoding performance. Therefore, the LSTM model was trained and evaluated separately for all 35 participants of the MyoKi database using all EMG and IMU data as input.

The second evaluation case investigated the contribution of different sensor modalities (EMG, FMG, ACC, and GYR) used as model input to decoding accuracy. To ensure fair comparison between modality combinations, the dimensionality of the input vector and the LSTM architecture were kept constant across all conditions. Modalities not included in a given configuration were replaced by zero-filled features, such that differences in performance could not be attributed to changes in model size or input dimensionality. This also resulted in identical computation times for each condition. The following input combinations were evaluated: EMG, FMG, EMG+GYR+ACC, FMG+GYR+ACC, EMG+FMG, EMG+FMG+ACC, EMG+FMG+GYR, EMG+FMG+ACC+GYR. Since FMG data were only available for a subset of participants in the MyoKi database, all analyses in this evaluation case were conducted exclusively on this FMG subset, including configurations that did not use FMG as input. This ensured that performance differences between modality combinations were not influenced by differences in participant composition. For each combination, models were trained and tested using the k-fold cross-validation protocol stated above. This allowed us to assess changes in $\textrm{R}^{2}$ for different combinations of modalities. We further analyzed which joints benefited most from specific input combinations regarding the $\textrm{R}^{2}$ score. For instance, we examined whether the inclusion of IMU or FMG data improved decoding of particular joints more than others, and considered whether biomechanical explanations could account for these differences.

The third evaluation case investigated the contribution of different anatomical regions to decoding accuracy. Input data were restricted to specific subsets of sensors using EMG and IMU data, allowing us to assess the relative importance of the muscle regions targeted by the multimodal bracelet (EMG channels 1 to 6 and corresponding IMU data), additional forearm sensors (EMG channels 7 and 8 with corresponding IMU data), and sensors on the upper arm (EMG channels 9 to 12 with corresponding IMU data). The following configurations were tested: Bracelet, Bracelet + Upper arm, Bracelet + Forearm, All sensors. As in evaluation case 1, this analysis was performed on all 35 participants using the same k-fold cross-validation protocol. As in evaluation case 2, removed channels were replaced with zeros to retain a fixed model size. When excluding one sensor region, both EMG and the corresponding IMU sensors were removed together to ensure consistency. Performance between each configuration was compared to evaluate the relative importance of each muscle region. The analysis further focused on identifying which joints were most affected by including specific regions. Therefore, the difference in $\textrm{R}^{2}$ score was calculated for each joint and added muscle region.

The statistical analyses of the three evaluation cases included both non-parametric and parametric tests, chosen based on data normality and experimental design. Normality of data distributions was first assessed using the Shapiro–Wilk test. When normality assumptions were violated (e.g., for comparisons across task repetitions or sensor modalities), Friedman tests were applied to evaluate overall differences between related samples, followed by Wilcoxon signed-rank tests for pairwise post-hoc comparisons, with Holm–Bonferroni or Benjamini-Hochberg FDR corrections to control for multiple comparisons. For data meeting normality assumptions (e.g., sensor layout comparisons), a repeated-measures ANOVA was used, with paired t-tests for post-hoc contrasts, again adjusted using Benjamini-Hochberg correction. Multiple linear regression (MLR) analyses were employed to examine relationships between decoding performance ($\textrm{R}^{2}$) and participant-related predictors, with collinearity checks performed using Pearson correlation and variance inflation factor (VIF). Forward selection guided by the Akaike information criterion (AIC) identified the best-fitting MLR models.

In evaluation cases 2 and 3, we further aimed to determine whether the benefit of adding input modalities or muscle regions to the sensor configuration depends primarily on the baseline decoding performance for each joint, or whether other factors (such as anatomical or sensor-specific properties) play a role. For this purpose, we calculated Pearson’s correlation coefficient between the baseline $\textrm{R}^{2}$ score and the improvement in $\textrm{R}^{2}$ score for each joint after adding sensor modalities or muscle regions. This analysis tests the hypothesis that joints with lower baseline decoding accuracy (i.e., lower $\textrm{R}^{2}$ with the simpler configuration) may experience greater improvements when additional modalities or muscle regions are included. If this relationship was not observed, we further identified the upper quartile of joints (the five joints with the greatest absolute improvement in $\textrm{R}^{2}$ score for each comparison), to potentially reveal anatomical or functional reasons underlying the observed improvements.

## Results

In the following, the results of three evaluation cases are presented that are designed to assess the impact of participant- and experiment-related factors (evaluation case 1), sensor modalitiy combinations (evaluation case 2), and muscle regions selection (evaluation case 3) on the performance of decoding biosignals from participants’ right arms into 18-channel hand and wrist kinematics using an LSTM network. For the evaluation, the MyoKi database was leveraged, which contains muscular activity and hand kinematics data from 35 participants performing 74 different tasks, each repeated six times. Depending on the evaluation case, the input data included up to 12-channel EMG, data from 9 IMUs (each comprising a 3-axis accelerometer and 3-axis gyroscope; 54 channels in total), and 24-channel FMG recorded around the upper forearm. The exact sensor layout is provided in the MyoKi database descriptor^45^, which also includes a detailed technical validation of data quality. Input data for the LSTM network was split by repetition into training, validation, and test sets. To account for variability across repetitions, we employed k-fold cross-validation. Model performance in decoding muscular activity into continuous hand and wrist motions was quantified using the coefficient of determination ($\textrm{R}^{2}$). Unless otherwise stated, reported $\textrm{R}^{2}$ scores represent overall decoding performance computed over the complete test repetition (containing all tasks) for each participant and cross-validation fold, and averaged across all decoded joints.

### Evaluation case 1: Exploring the impact of participant- and experiment-related factors

#### Effects of muscle fatigue and repetition duration on decoding performance

**Fig. 2:**
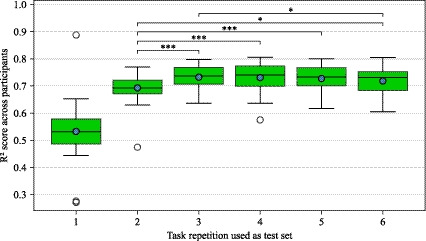
Overall $\textrm{R}^{2}$ scores across participants for each task repetition used as the test set in the repetition-based cross-validation. Each repetition corresponds to one full execution of the complete task protocol (see "Methods"), and in each cross-validation fold, one repetition served as the test set. All pairwise comparisons with repetition 1 as the test set using the Wilcoxon signed-rank test are significant, but are left out for better readability. Holm–Bonferroni correction was used to account for multiple comparisons and reduce the risk of Type I errors. Asterisks indicate significance levels (p < 0.05: *, p < 0.01: **, p < 0.001: ***). Across participants, the lowest $\textrm{R}^{2}$ values were obtained when repetition 1 was used as the test set, whereas repetitions 3 to 6 yielded the highest performance.

To test for the influences of muscle fatigue and recording time of the test repetition, decoding performances of each repetition across participants were compared, with results shown in Fig. 2. We first assessed the normality of the differences between each pair of task repetitions using the Shapiro–Wilk test. As the assumption of normality was not met, a Friedman test was performed, which revealed a significant effect of the task repetition used as test set on the $\textrm{R}^{2}$ score across participants ($\chi ^2=94.46$, p < 0.001) with a large effect size (Kendall’s $W=0.54$). Post-hoc pairwise comparisons using the Wilcoxon signed-rank test with Holm-Bonferroni correction showed that all repetitions compared to repetition 1 yielded significantly higher $\textrm{R}^{2}$ scores (p < 0.05). Among the remaining repetitions, only a subset of pairwise comparisons reached significance (see Fig. 2).

**Fig. 3:**
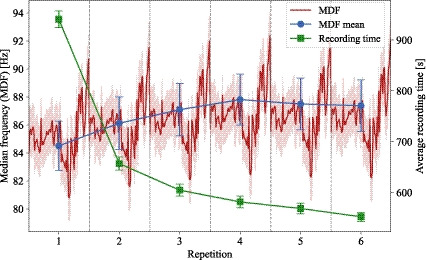
Participant-averaged median frequency (MDF) and recording time across repetitions and tasks. The red line shows MDF over tasks and repetitions averaged across all participants, with shaded areas representing the standard error across participants. Blue circles indicate the mean MDF per repetition, with error bars representing the standard error across participants. The green line represents the average recording time per repetition averaged across participants, with error bars indicating the standard error across participants. Results illustrate that MDF is strongly task dependent and only slightly increases across repetitions, while recording time systematically decreases for later repetitions due to habituation effects of the participants with the performed tasks. We found a very strong negative correlation between normalized recording time and fatigue across repetitions (r = -0.96)..

Multiple linear regression (MLR) analyses were conducted to assess how recording time and fatigue affect decoding performance. Fatigue, a key factor influencing EMG signal quality, was quantified using the median frequency (MDF), where higher values indicate less fatigue. Initial collinearity assessment revealed a very strong negative correlation between normalized recording time and fatigue across repetitions (r = -0.96) as indicated in Fig. 3. Variance Inflation Factor (VIF) analysis confirmed strong collinearity, with predictors exhibiting VIF values of 12.13, far exceeding the commonly accepted threshold of 5. Therefore, each predictor was tested independently. This separate-model analysis served as a sensitivity analysis to assess whether the observed effects remained consistent despite the strong collinearity between recording time and MDF. The model using normalized recording time as the sole predictor yielded the lowest Akaike information criterion (AIC), indicating the best fit. In this model, the recording time of the repetition used as the test set had a significant negative association with $\textrm{R}^{2}$ (coefficient = -0.0768, p < 0.001), meaning that repetitions with longer recording times used as the test set led to lower $\textrm{R}^{2}$ scores. In contrast to the high collinearity between MDF and recording time per repetition, collinearity analysis indicated a low correlation between recording time and MDF for each individual task (r = 0.17). Figure 3 already indicates that MDF is vastly task dependent and only slightly changes across repetitions. To properly assess reproducibility of MDF across repetitions, intraclass correlation coefficients (ICC) was computed, treating tasks as targets and repetitions as repeated ratings. ICC indicates how much of the total variability reflects true between-task differences rather than repetition-related noise. Single-repetition reliability was excellent (ICC(2,1) = 0.896), and reliability of the repetition-averaged MDF was near-perfect (ICC(2,6) = 0.981). Together with the near-unity rank correlations between repetitions, this indicates that MDF is strongly task-dependent in this database, with comparatively small repetition-related variation.

#### Multicollinearity analysis of participant-related factors

**Fig. 4:**
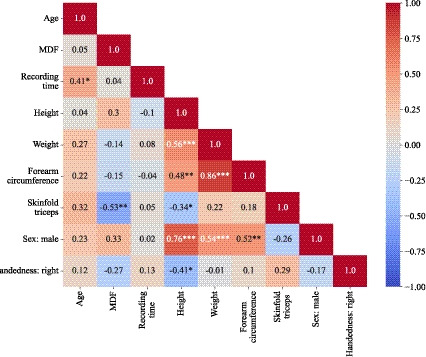
Correlation matrix of continuous and categorical predictors represented by Pearson correlation coefficients. Height is strongly associated with weight, forearm circumference, and sex, while weight also correlates with forearm circumference and sex. Age shows a significant positive correlation with recording time. Asterisks indicate significance levels (p < 0.05: *, p < 0.01: **, p < 0.001: ***).

Prior to regression modeling, correlations and potential multicollinearity among participant- and experiment-related predictors were analyzed. Figure 4 displays the correlations among all investigated variables as a heat map. MDF serves as a robust indicator of muscle fatigue, capturing physiological changes that may not be reflected by the participant-related measures alone, and was thus included for each participant as an additional predictor. The results show a significant negative correlation between MDF and skinfold thickness at the triceps (p < 0.01), suggesting a link between the EMG frequency spectrum and local body fat. Height is strongly correlated with weight (p < 0.001), forearm circumference (p < 0.01), and sex (p < 0.001), reflecting expected anthropometric relationships. Weight was also significantly correlated with forearm circumference (p < 0.001) and sex (higher for men, p < 0.001). Furthermore, higher age significantly correlates with longer recording times (p < 0.05).

#### Factors predicting participant-wise decoding performance

After assessing correlations and multicollinearity among predictors, multiple linear regression analyses were performed to investigate which participant-related factors best explain overall $\textrm{R}^{2}$ scores across participants. All continuous predictors (age, MDF, recording time, height, weight, forearm circumference, skinfold at triceps) were standardized (z-scored) prior to analysis. Categorical variables (sex and handedness) were dummy-coded. To identify the best set of predictors for explaining the variance in the overall $\textrm{R}^{2}$ score across participants, we performed forward selection of predictors guided by the AIC score. At each step, the predictor that most improved the model fit was added. Models with an AIC difference of less than 2 were considered to have equivalent fit to the data. The final model included MDF and weight with detailed results summarized in Table 3. Both MDF (p < 0.01) and weight (p < 0.05) were statistically significant predictors.

**Table 3:** Best-fitting multiple linear regression model after forward selection using participant-related factors predicting $\textrm{R}^{2}$ across participants of the MyoKi database. The table reports regression coefficients, standard errors (SE), t-values, p-values, and the 2.5% and 97.5% columns indicate the lower and upper bounds of the 95% confidence interval for each coefficient estimate.

	Coef.	SE	t	p	2.5%	97.5%
Intercept	0.6890	0.006	120.264	0.000	0.677	0.701
MDF	0.0179	0.006	3.096	0.004	0.006	0.030
Weight	-0.0146	0.006	-2.528	0.017	-0.026	-0.003

#### Joint-wise decoding performance

**Fig. 5:**
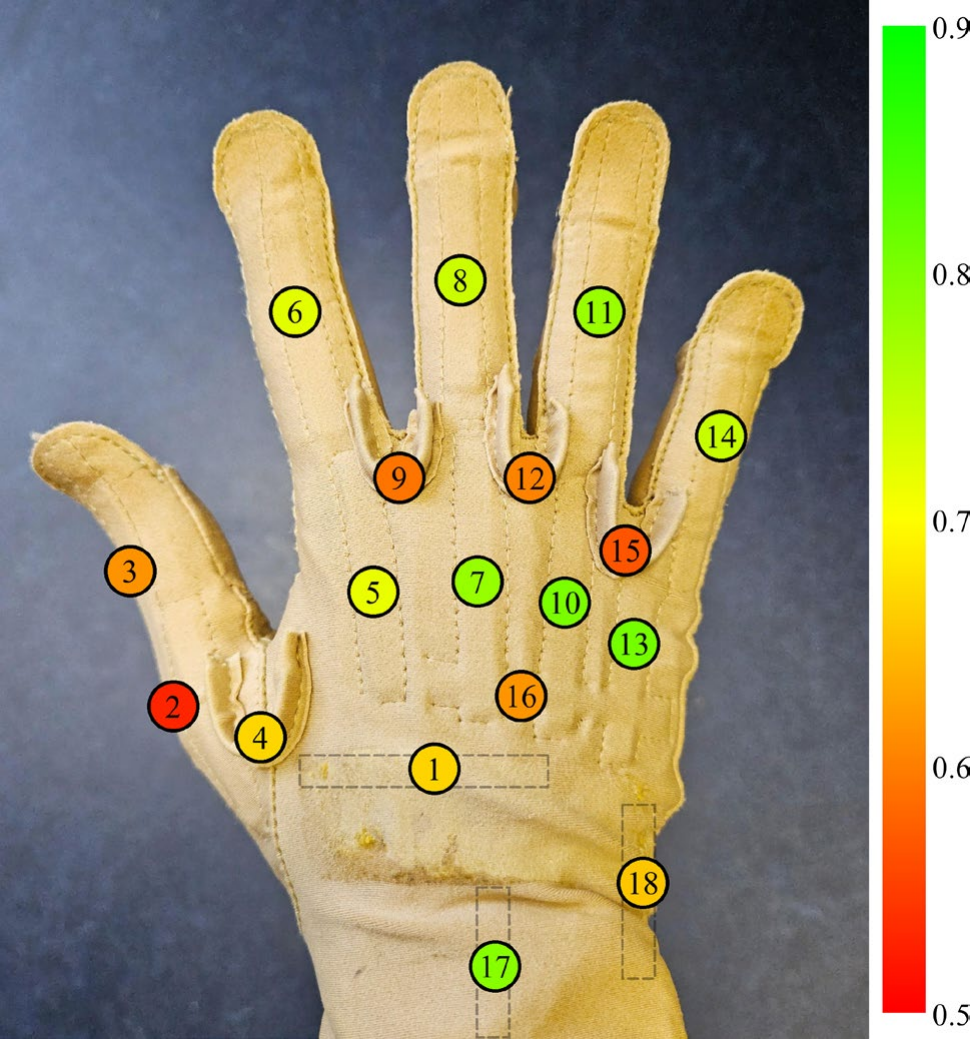
Heat map of joint-wise $\textrm{R}^{2}$ scores averaged across all participants and cross-validation folds, using EMG and IMU data as inputs to the LSTM network for decoding hand kinematics. Colors represent prediction accuracy (red = lower $\textrm{R}^{2}$, green = higher $\textrm{R}^{2}$), with numbered circles corresponding to individual CyberGlove sensors. On average, highest $\textrm{R}^{2}$ scores were observed for wrist flexion/extension, and metacarpophalangeal (MCP) and proximal interphalangeal (PIP) joints of the index, middle, ring, and little fingers.

Fig. 5 shows the average decoding performance ($\textrm{R}^{2}$ scores) across 35 participants of the MyoKi database for each CyberGlove sensor. The highest accuracies were observed for the MCP joints of the index, middle, ring, and little fingers (sensors 5, 7, 10, and 13), the PIP joints (sensors 6, 8, 11, and 14), and wrist flexion/extension (sensor 17), with $\textrm{R}^{2}$ scores ranging from 0.716 (sensor 5) to 0.808 (sensor 13). In contrast, the thumb MCP and IP joints (sensors 2 and 3), finger adduction/abduction of the index, middle, ring, and little fingers (sensors 9, 12, and 15), and the palmar arch (sensor 16) showed much lower accuracies, with $\textrm{R}^{2}$ scores between 0.526 (sensor 2) and 0.617 (sensor 16).

### Evaluation case 2: Sensor modality combinations

**Fig. 6:**
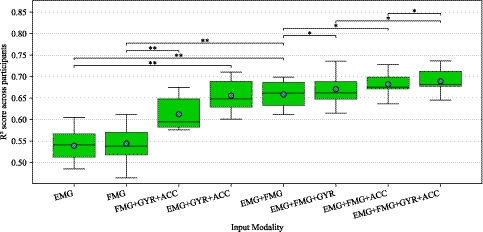
Comparison of $\textrm{R}^{2}$ scores across participants for different input modalities from the multimodal bracelet. Each box represents the $\textrm{R}^{2}$ scores across participants for a given modality, sorted by mean $\textrm{R}^{2}$ from left to right. For each participant, $\textrm{R}^{2}$ scores were averaged across all folds. The boxes represent the interquartile range, with the horizontal line indicating the median and the blue circle marking the mean value for each layout. Whiskers extend to the most extreme data points not considered outliers, and outliers are shown as individual points. Significance bars indicate statistically significant differences between selected modality pairs (Wilcoxon signed-rank test with Benjamini-Hochberg FDR correction) with asterisks indicating significance levels (p < 0.05: *, p < 0.01: **). The plot shows significant improvements in $\textrm{R}^{2}$ for each added modality, with the highest accuracy achieved when all modalities were combined. All significant comparisons showed large effects, as indicated by the rank-biserial correlation (|*r*| > 0.74).

We compared the overall decoding performance ($\textrm{R}^{2}$ scores) of different sensor modalities across 10 participants from the second subset of the MyoKi database, which includes FMG data. The modalities included EMG, FMG, FMG+GYR+ACC, EMG+GYR+ACC, EMG+FMG, EMG+FMG+GYR, EMG+FMG+ACC, and EMG+FMG+GYR+ACC. The distribution of $\textrm{R}^{2}$ scores for each modality is shown in Fig. 6. Normality testing (Shapiro–Wilk) indicated that at least one modality was not normally distributed, so the Friedman test was used for overall comparison, which revealed a significant effect of input modality on $\textrm{R}^{2}$ scores ($\chi ^2=57.53$, p < 0.001) with a large effect size (Kendall’s $W=0.82$). Post-hoc pairwise comparisons between selected modality pairs were performed using the Wilcoxon signed-rank test with Benjamini-Hochberg FDR correction. Significant differences of pairwise comparisons are indicated by asterisks in Fig. 6. All significant comparisons showed large effects, as indicated by the rank-biserial correlation (|*r*| > 0.74).

Averaged across participants, decoding accuracy ($\textrm{R}^{2}$ score) improved for all joints with each additional sensor modality. For the comparison between EMG+FMG and EMG, a moderate negative correlation was observed between the baseline $\textrm{R}^{2}$ scores using EMG only and the improvement in $\textrm{R}^{2}$ scores after adding FMG (Pearson r = -0.433, p = 0.0724). However, this result did not reach statistical significance, indicating that the benefit from FMG was not strongly related to baseline joint performance with EMG alone. For all other comparisons, the negative correlation between the $\textrm{R}^{2}$ improvement and the baseline $\textrm{R}^{2}$ scores was statistically significant. Specifically, for EMG+FMG vs FMG (Pearson r = -0.495, p = 0.0369), EMG+GYR+ACC vs EMG (Pearson r = -0.583, p = 0.0112), FMG+GYR+ACC vs FMG (Pearson r = -0.816, p < 0.001), EMG+FMG+GYR vs EMG+FMG (Pearson r = -0.672, p = 0.0022), and EMG+FMG+ACC vs EMG+FMG (Pearson r = -0.560, p = 0.0156), joints with lower baseline decoding accuracy for the simpler modality tended to benefit more from the addition of further sensor modalities. For sensor modality comparison of EMG+FMG vs EMG, where the hypothesized relationship was not met, we further identified the upper quartile of joints, which are the five joints with the greatest absolute improvement in $\textrm{R}^{2}$ score. For the EMG+FMG vs EMG comparison, the CyberGlove channels 1 (+0.139), 3 (+0.140), 4 (+0.148), 6 (+0.139), and 8 (+0.141) exhibited the largest improvements in $\textrm{R}^{2}$, potentially reflecting anatomical or functional factors influencing sensor effectiveness.

### Evaluation case 3: muscle region selection

**Fig. 7:**
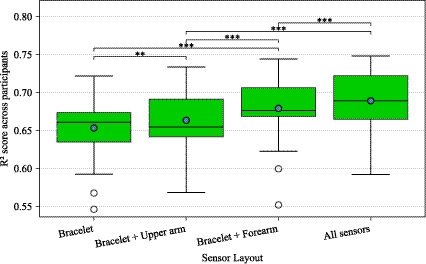
Box plots showing the distribution of $\textrm{R}^{2}$ scores across participants for four sensor layouts: Bracelet, Bracelet + Upper arm, Bracelet + Forearm, and All sensors. For each participant, one $\textrm{R}^{2}$ score was computed per cross-validation fold using the complete test repetition containing all tasks, and then averaged across all six folds and joints. The boxes represent the interquartile range, with the horizontal line indicating the median and the blue circle marking the mean value for each layout. Whiskers extend to the most extreme data points not considered outliers, and outliers are shown as individual points. Statistically significant differences between selected pairs of sensor layouts are indicated by bars and asterisks above the plot (paired t-test, Benjamini-Hochberg FDR correction), with asterisks indicating significance levels (p < 0.01: **, p < 0.001: ***). The decoding performance using only the multimodal bracelet sensors is used as the reference. The remaining sensor layouts are displayed along the x-axis, ordered by their mean $\textrm{R}^{2}$ scores. Overall, more sensors lead to higher decoding accuracy, with the largest improvement in $\textrm{R}^{2}$ coming from the additional forearm sensors. Effect sizes relative to the Bracelet condition were small to medium for Bracelet + Upper arm (Cohen’s $d_z = 0.48$), large for Bracelet + Forearm ($d_z = 0.89$), and very large for All sensors ($d_z = 1.09$)..

Four different sensor layouts, covering different muscle regions, were compared by evaluating overall $\textrm{R}^{2}$ scores computed for the complete test repetition of each fold and averaged across all six cross-validation folds and all joints. Figure 7 shows the $\textrm{R}^{2}$ scores across participants for each sensor layout: Bracelet (0.6642 ± 0.0572), Bracelet + Upper arm (0.6832 ± 0.0582), Bracelet + Forearm (0.6902 ± 0.0572), and All sensors (0.7042 ± 0.0587). Normality was confirmed for all sensor layouts using the Shapiro–Wilk test. A repeated-measures ANOVA revealed a significant effect of sensor layout on $\textrm{R}^{2}$ scores (F = 5.51, p = 0.0013) with a medium to large effect size ($\eta ^{2}_{p}=0.14$). Post-hoc paired t-tests with Benjamini-Hochberg FDR correction identified significant differences between all pairs.

Averaged across participants, $\textrm{R}^{2}$ scores improved for all joints with each additional muscle region. Effect sizes relative to the Bracelet condition were medium for Bracelet + Upper arm (Cohen’s $d_z = 0.48$), large for Bracelet + Forearm ($d_z = 0.89$), and very large for All sensors ($d_z = 1.09$). For the comparison between Bracelet + Upper arm and Bracelet, a significant negative correlation was observed between the baseline $\textrm{R}^{2}$ scores (Bracelet only) and the improvement in $\textrm{R}^{2}$ scores after adding upper arm sensors (i.e., the difference between Bracelet + Upper arm and Bracelet for each joint; Pearson r = -0.635, p = 0.0047). This indicates that joints with lower baseline decoding accuracy tended to benefit more from the inclusion of upper arm sensors. In contrast, the correlation between the $\textrm{R}^{2}$ improvement from adding forearm sensors (Bracelet + Forearm minus Bracelet) and the baseline $\textrm{R}^{2}$ scores (Bracelet only) was weaker and not statistically significant (Pearson r = -0.331, p = 0.1796), suggesting that the benefit from forearm sensors was not strongly related to baseline joint performance. Since the hypothesized relationship was not met for the addition of forearm sensors, we further identified the upper quartile of joints (the five CyberGlove channels with the greatest absolute improvement in $\textrm{R}^{2}$ score for this comparison). The channels 2 (+0.034), 3 (+0.033), 4 (+0.028), 5 (+0.038), and 18 (+0.039) exhibited the largest improvements, potentially reflecting anatomical factors influencing sensor effectiveness.

## Discussion

The results from Fig. 2 indicate that the choice of test repetition substantially influences model performance, particularly when using the first repetition as the test set. Multiple linear regression analysis revealed a significant negative association between the recording time of the repetition used as the test set and the $\textrm{R}^{2}$ score, with longer recording times leading to reduced decoding accuracy. This effect can be explained by two factors. First, using a longer repetition as the test set reduces the amount of data available for training, which limits model generalizability, and second, longer repetitions may encompass greater variability in movement execution, increasing decoding difficulty. Factors such as participant fatigue, signal drift, or changes in muscle activation patterns over time may contribute to this decline in accuracy. Collinearity assessment further revealed a strong negative correlation between normalized recording time and fatigue across repetitions (r = -0.96, VIF = 12.13), as shown in Fig. 3, with muscle fatigue decreasing for later repetitions (indicated by higher MDF). Typically, muscle fatigue would be expected to accumulate over time, especially without proper inter-trial breaks. However, the potential short breaks during the transition between two trials or between repetitions, combined with a reduction in recording duration due to habituation effects, may explain the observed pattern. Nevertheless, the repeated-trial design may still have influenced fatigue development across the recording session and should therefore be considered when interpreting the results. Due to the strong collinearity between recording time and MDF across repetitions, predictors were tested independently, and the model using normalized recording time as the sole predictor yielded the lowest AIC, suggesting that recording time provides the most consistent explanation of the observed reduction in model performance.

When analyzing data at the level of individual tasks, the correlation between recording time and MDF was low (r = 0.17), indicating that muscle fatigue as measured by MDF is more dependent on the specific task than on the uninterrupted accumulation of active muscle activation time alone. Figure 3 further illustrates that MDF varies substantially between tasks and changes only slightly across repetitions, consistent with the high intraclass correlation coefficients (ICC) across repetitions, supporting strong task dependence of MDF. These findings, consistent with prior evidence, emphasize that muscle fatigue is not a uniform process but is likely influenced by the type of movement performed and the required force. The task-specific differences in MDF suggest that the nature of the muscle activation during each task plays a more important role in fatigue and may contribute significantly to performance variability across tasks, which needs to be tested in future experiments.

The multiple linear regression analysis of participant-related factors (Table 3) revealed reduced $\textrm{R}^{2}$ values for participants with lower MDF in the EMG signal. Although MDF is commonly interpreted in the context of muscle fatigue, it is not specific to fatigue alone, which introduces more variability and noise into the muscle signals^21,22^. As MDF is derived from the surface EMG power spectrum, it is influenced by muscle fiber conduction velocity, motor unit discharge behavior, and volume conductor properties, including subcutaneous tissue and electrode-skin interface characteristics^60^. Accordingly, the association between lower MDF and reduced decoding performance likely reflects a combination of physiological state and signal transmission characteristics rather than fatigue in isolation. Higher body weight was also associated with reduced $\textrm{R}^{2}$, consistent with the notion that increased subcutaneous adipose tissue attenuates and spatially smooths surface muscle signals. However, triceps skinfold thickness did not significantly predict $\textrm{R}^{2}$, suggesting that body weight may reflect broader body composition or distributed tissue properties not captured by a single local measure, or that local subcutaneous fat tissue alone is insufficient to explain differences in signal quality. Overall, MDF and body weight should be interpreted as composite markers reflecting task-dependent neuromuscular activation, physiological state, and signal transmission characteristics, which jointly contribute to variability in decoding performance. A more detailed analysis of inter-channel relationships was not pursued, given the uniform, mostly non-muscle-specific sensor layout of the MyoKi database, which limits the physiological interpretability of channel-wise connectivity measures, but could be considered in future work with anatomically targeted recordings. The interrelationships between some of the participant-related factors illustrated in Fig. 4 highlight the importance of considering multicollinearity when interpreting regression results, as some predictors may capture overlapping physiological information. Sex, for instance, has no statistically significant impact on the decodability of muscular activity into hand motions. However, it should be noted that sex correlates with weight (r = 0.54, p < 0.001), which is higher for men and significantly influences decodability. Further, the multicollinearity matrix from Fig. 4 shows that higher age correlates with longer recording times, indicating that older participants tended to require more time for task completion. Negative age-related impacts on muscle signals and thus the $\textrm{R}^{2}$ score could be offset by the benefits of longer recording times and more data, which could otherwise hide significant impacts. Additional participant-specific factors not available in the dataset, such as exercise habits, upper-limb muscular development, or skin type, may further influence sEMG and FMG signal characteristics and contribute to inter-subject variability in decoding performance. While some aspects related to tissue composition are partially reflected by participant-related measures such as forearm circumference and skinfold thickness, future datasets should include more comprehensive physiological and lifestyle-related information.

**Fig. 8:**
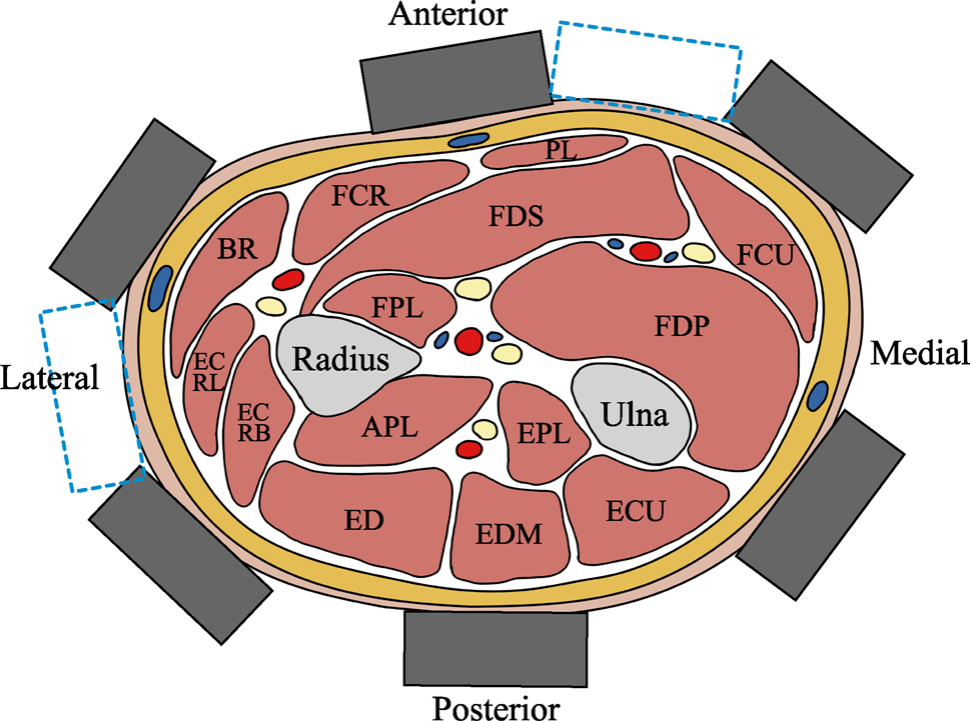
Cross-sectional anatomy of the right forearm at the level of the proximal third, illustrating major extrinsic muscles relevant for hand and wrist movements. The positions of the surface EMG electrodes of the multimodal bracelet are shown as gray rectangles, and the additional EMG sensors at the lower/mid forearm are displayed as blue dashed rectangles. Muscle abbreviations: BR = brachioradialis, FCR = flexor carpi radialis, PL = palmaris longus, FCU = flexor carpi ulnaris, FDS = flexor digitorum superficialis, FDP = flexor digitorum profundus, FPL = flexor pollicis longus, APL = abductor pollicis longus, EPL = extensor pollicis longus, ED = extensor digitorum, EDM = extensor digiti minimi, ECRL = extensor carpi radialis longus, ECRB = extensor carpi radialis brevis, ECU = extensor carpi ulnaris. These muscles can be grouped into deep extrinsic muscles (FPL, EPL, and APL) and superficial extrinsic muscles (all remaining muscles)..

The results from Fig. 5 show large differences in average $\textrm{R}^{2}$ scores across participants between joints. The observed performance differences across joints can likely be attributed to the anatomical distribution of the muscles (see Fig. 8) that control these movements. Those muscles can be grouped into deep extrinsic muscles (FPL, EPL, and APL), superficial extrinsic muscles (all remaining forearm muscles shown in Fig. 8), and intrinsic hand muscles that are not covered by the sensor layout, corresponding to decreasing levels of surface EMG and FMG accessibility. However, anatomical accessibility is likely not the only factor influencing decoding performance. Differences in movement variability across joints, as well as potential differences in hand kinematic measurement accuracy and signal-to-noise ratio, may also contribute to the observed $\textrm{R}^{2}$ differences. The metacarpophalangeal (MCP) and proximal interphalangeal (PIP) joints of the index, middle, ring, and little fingers, which achieved the highest decoding accuracies, are controlled primarily by the flexor digitorum superficialis (FDS), flexor digitorum profundus (FDP), and extensor digitorum (ED)^61^. These extrinsic finger muscles are located superficially in the forearm and are well covered by the EMG electrode placement described in the MyoKi data descriptor^45^, which is also shown in Fig. 8. Similarly, wrist flexion (driven by flexor carpi radialis (FCR) and flexor carpi ulnaris (FCU)) and wrist extension (driven by extensor carpi radialis longus (ECRL), extensor carpi radialis brevis (ECRB), and extensor carpi ulnaris (ECU)) rely on superficial forearm muscles^61^, which explains the high $\textrm{R}^{2}$ scores observed for wrist kinematics.

By contrast, the thumb MCP and interphalangeal (IP) joints are controlled either by intrinsic hand muscles (e.g., flexor pollicis brevis, extensor pollicis brevis) that lie outside the recording region, or by deep extrinsic muscles (flexor pollicis longus (FPL), extensor pollicis longus (EPL))^61^ that are less accessible to surface recordings. Similarly, palmar arching and finger abduction/adduction depend mainly on intrinsic hand muscles such as the interossei^61^, which explains their lower decoding accuracy. Overall, joints controlled primarily by superficial extrinsic forearm muscles achieved higher decoding performance than joints relying on deep or intrinsic muscles. At the same time, certain thumb and abduction/adduction movements may inherently exhibit greater kinematic variability across repetitions and participants, which could further reduce achievable decoding performance. In addition, glove-based measurements for these joints may be more susceptible to noise and fitting-related inaccuracies due to their smaller ranges of motion and more complex movement patterns. Therefore, the presented anatomical interpretation should be regarded as a plausible contributing explanation rather than the sole determinant of decoding accuracy.

From a physiological standpoint, this anatomical distinction suggests a fundamental limitation of forearm EMG-based interfaces. Although movements involving extrinsic muscles can be decoded with relatively high accuracy ($\textrm{R}^{2}$ up to 0.808), fine motor tasks driven by intrinsic muscles remain challenging. In robotic teleoperation, this limitation can often be mitigated by integrating additional sensors targeting these intrinsic muscles. However, it poses a more serious problem in prosthetic applications. While deep extrinsic muscles can here be accessed through complementary sensing modalities (e.g., intramuscular EMG, ultrasound)^62,63^, there is often no option to place additional sensors at the lower forearm or hand due to limb loss. Although the MyoKi database does not include data from individuals with transradial amputation, the present results highlight fundamental limitations of forearm-based decoding of muscular activity into continuous hand motions. Transradial amputees could, in principle, replicate the tasks in the MyoKi database by recording hand kinematics from the intact hand and muscular activity from the residual limb during bilateral execution, where the intact hand mimics the phantom movements of the missing hand, as shown in previous work^33,64,65^. However, the absence of signals from intrinsic hand muscles remains a critical challenge. Researchers should recognize that even the most advanced decoding models are unlikely to overcome this limitation when relying solely on muscular activity, as the data required to fully reconstruct continuous and realistic hand motions are simply missing. One straightforward solution would be to restrict control to the most reliable joints, but this comes at the cost of severely reduced functionality. In particular, thumb control, which depends heavily on intrinsic muscles, is essential for most daily tasks, and adduction/abduction movements would also be lost. Novel approaches are therefore needed. Recent studies have investigated decoding hand motion directly from peripheral nerve signals, including those that would normally innervate the intrinsic hand muscles^66–68^, including a recent review highlighting this potential^69^. While nerve signals are small and subject to interference (amplitude in the 5–20 $\mu$V range)^70^, current nerve-based decoding has the potential to provide critical information for joints that rely on intrinsic muscles^67^. Incorporating these signals could ultimately improve control accuracy to a level sufficient for everyday use^69^. Since in prosthetic applications, full hand functionality cannot be achieved through muscular activity-based decoding alone, we propose that future research should pursue hybrid approaches that combine muscular activity from the forearm with nerve signals corresponding to the missing intrinsic muscles.

The comparison of $\textrm{R}^{2}$ scores across participants for different sensor modalities used as model input demonstrates that combining multiple sensor modalities significantly enhances model performance compared to using single modalities alone. Notably, EMG and FMG as standalone modalities did not differ significantly in their baseline decoding accuracy, suggesting that both provide comparable information for continuous hand motion decoding. It is important to note, however, that the multimodal bracelet used in this study included 24 FMG channels but only 6 EMG channels, which may influence decoding performance for each modality. Interestingly, the addition of FMG data to EMG did not primarily benefit joints with the lowest baseline $\textrm{R}^{2}$ scores. Instead, the most pronounced improvements were observed for joints (channels 1, 3, 4, 6, and 8 from Fig. 5) associated with specific deep extrinsic muscles, such as abductor pollicis longus (APL) for thumb abduction (channel 4), FPL for thumb IP flexion (channel 3), and FDS, FDP, and ED for flexion and extension of the PIP joints of the index and middle fingers (channels 6 and 8)^61^. Many of these muscles, particularly the FDS, FPL, and APL, are located deep within the forearm^61^ (see Fig. 8). Both surface EMG and FMG are primarily sensitive to superficial muscle activity and are not well suited to directly capture signals from deep muscles. However, the two modalities capture fundamentally different physiological information. EMG measures the electrical activation of muscles, whereas FMG captures pressure and volumetric changes at the skin surface caused by muscle contractions. Consequently, FMG may provide complementary mechanical information related to tendon displacement, muscle swelling, and distributed tissue deformation that is not directly represented in EMG signals. Therefore, it is notable that improvements in $\textrm{R}^{2}$ scores were observed for joints controlled by these deep extrinsic muscles. This complementary sensing principle, combined with the higher spatial density of FMG channels (four FMG channels for each EMG channel), may provide richer multidimensional information about muscle contractions, potentially capturing subtle surface deformations^54,55^ indirectly related to the activity of deeper muscles. Although the dimensionality of the input vectors and the LSTM architecture were kept constant across modality conditions through zero-padding of unused channels in the feature vector, the denser spatial coverage provided by FMG sensors may still contribute to improved decoding performance independently of physiological modality differences. In contrast, when EMG data was added to FMG, the improvement in $\textrm{R}^{2}$ showed a significant negative correlation with the baseline $\textrm{R}^{2}$ of FMG alone. This indicates that joints with lower baseline decoding accuracy using FMG benefited most from the addition of EMG. This finding suggests that FMG already provides informative complementary mechanical information across many joints, while EMG contributes complementary information about neural muscle activation patterns^49^, particularly for movements that are otherwise difficult to distinguish using pressure-based sensing alone. For all other modality comparisons, a significant negative correlation was found between $\textrm{R}^{2}$ improvement and baseline $\textrm{R}^{2}$ across joints. This suggests that the benefit of adding EMG, GYR, or ACC as input modalities is generally greater for joints that are more challenging to decode with the baseline modality alone. In particular, ACC signals may provide complementary biomechanical information related not only to gross limb acceleration, but also to subtle soft-tissue oscillations and muscle vibrations generated during muscle contractions^51^ These vibration patterns may indirectly reflect activation levels of specific muscle regions and therefore provide information that is not directly represented in EMG or FMG signals. Similarly, GYR signals provide information about forearm and wrist orientation during movement. This information may help compensate for changes in EMG and FMG signal characteristics caused by arm-position-dependent muscle deformation and sensor shift. Since changes in forearm orientation can alter muscle geometry, tendon displacement, and electrode-skin contact conditions, orientation information from gyroscopes may provide contextual information that improves decoding robustness across varying arm postures and dynamic movements.

The combination of all four modalities yielded the highest overall $\textrm{R}^{2}$ scores and showed significant improvements over all other modality combinations. The most substantial gains were observed when EMG was combined with either inertial sensor data (GYR, ACC) or FMG, as reflected by the significant differences in $\textrm{R}^{2}$ scores for these pairs (see Fig. 6). The combination of EMG and inertial sensor data is particularly promising, given that many commercial EMG sensors already integrate IMUs. While FMG sensors are not yet widely used in commercial hand prostheses, the present results suggest they could be a valuable addition. However, practical challenges remain for FMG implementation, such as ensuring reliable force transfer when applied to a prosthesis socket. Overall, these findings highlight the value of multimodal sensor fusion, particularly for joints with low baseline decoding performance. The choice of sensor modality (EMG, FMG, GYR, ACC) has an impact on decoding accuracy, with the addition of each sensor modality leading to significantly higher decoding accuracies. It should be noted, however, that increasing the amount of input data also raises computational cost and, consequently, power consumption, which is an important consideration in the design of wearable devices such as prostheses.

The comparison of $\textrm{R}^{2}$ scores across participants for different muscle regions used as model input demonstrates that expanding sensor coverage to each additional muscle region leads to significant improvements in continuous hand motion decoding. Notably, the inclusion of upper arm sensors alongside the bracelet sensors on the upper forearm resulted in a significant increase in decoding accuracy. Since the neural network architecture and model size were held constant by replacing removed channels with zeros, as described in the "Methods" section, this improvement must be attributed to the unique information provided by the upper arm sensors. Although these electrodes do not directly target muscles responsible for hand and wrist motion, they may capture valuable contextual data. For instance, the upper arm IMU can provide information about arm orientation, while increased muscle activation in the upper arm may reflect the manipulation of heavier objects. Such contextual cues could help the model distinguish between free hand movements and object manipulation, the latter typically restricting hand motion due to contact forces. The improvement in $\textrm{R}^{2}$ from adding upper arm sensors was further significantly correlated with the baseline $\textrm{R}^{2}$ scores (bracelet only) across joints. This suggests that joints with lower baseline $\textrm{R}^{2}$ benefit most from the additional orientation and load-related information provided by the upper arm sensors, as these factors likely contributed to the reduced decoding accuracy of those joints when relying on forearm signals alone.

The addition of extra forearm sensors, indicated by blue dashed rectangles in Fig. 8, resulted in even greater improvements in decoding accuracy. Notably, this enhancement does not show a clear dependence on baseline joint decoding performance, suggesting that the benefit extends beyond joints with initially low $\textrm{R}^{2}$ scores. These additional forearm sensors are strategically positioned to capture signals from extrinsic muscles directly responsible for joint movements, such as PL, FDS, FCU, BR, ECRL, and ECRB. These muscles contribute to a variety of hand and wrist actions, including ulnar and radial deviation (channel 18 in Fig. 5) and MCP flexion of the index finger (channel 5)^61^. Although deeper muscles like FPL and APL may not be directly accessible to surface sensors, the placement of the additional forearm electrodes may allow for indirect detection of their activity, thereby enhancing decoding performance for movements such as IP flexion (channel 3) and thumb abduction (channel 4).

Using all available sensors again shows a significant improvement compared to using the bracelet and the additional forearm sensors. This highlights that the sensors on the upper arm do contain distinct information that is not present in the forearm sensors, such as additional orientation and load-related information. However, although this improvement reached statistical significance and was associated with a small to medium effect size, the average increase in decoding accuracy was minor ($\Delta$ $\textrm{R}^{2}$ = 0.0102). In practical applications such as prosthetic control, it remains unclear whether such a modest improvement would translate into perceptible improvements in usability, controllability, or user experience during daily tasks. Considering this modest improvement in decoding accuracy, it is important to consider whether additional sensors on the upper arm are justified for daily use, especially since sensors not integrated into the prosthesis socket may introduce additional limitations and sources of error in practical applications. Since additional sensors outside the prosthesis socket may also increase setup complexity and reduce comfort, the practical benefit of upper arm sensors must be carefully weighed against these drawbacks. The two additional forearm sensors, however, yield a substantially greater improvement in decoding accuracy, with an average $\textrm{R}^{2}$ increase of 0.0259 compared to the bracelet alone. This improvement was additionally associated with a large effect size, suggesting that the added forearm sensors provide a practically more relevant benefit for decoding performance. Therefore, incorporating these forearm sensors appears especially beneficial for robotic hand control, but also for prosthetic applications, provided that the residual limb and the design of the prosthesis socket permit their placement.

For reliable real-world hand motion decoding, additional engineering and clinical constraints must be considered that were beyond the scope of the present work. In practical prosthetic and teleoperation applications, model complexity directly affects computational delay, memory requirements, and power consumption, all of which are critical for wearable systems with limited onboard processing capabilities and battery capacity. Consequently, the number of biosignals used as input, the amount of extracted features, as well as the neural network architecture and model size, must be carefully balanced against real-time requirements and energy efficiency. In addition to these engineering constraints, physiological differences among users can substantially influence the robustness of decoding. This is particularly relevant for individuals with transradial amputation, where the anatomy and skin condition of the residual limb can vary considerably depending on the cause of amputation and surgical procedure^71^. Physiological characteristics of the residual limb, including scar tissue, changes in muscle anatomy, variations in soft tissue composition, and skin-related factors, can influence EMG signal acquisition and the stability of the electrode–skin interface^72^. In addition, practical challenges such as electrode displacement and sensor shift during everyday use may reduce decoding accuracy and compromise the long-term reliability of myoelectric control systems^73^. All analyses in this study were performed under a within-subject evaluation protocol. Therefore, the reported relationships between participant-related factors and decoding performance cannot be directly generalized to cross-subject transfer scenarios, which remain an important topic for future investigation.

The key findings of this work are summarized as follows:

- Participant-related factors such as the median frequency of the EMG signal (an indicator of muscle fatigue) and body weight significantly affect decoding performance of continuous hand and wrist motions across individuals.
- Joints primarily controlled by intrinsic muscles are particularly difficult to decode.
- Expanding sensor coverage to include additional muscle regions leads to significant improvements in decoding performance, highlighting the importance of spatial sensor distribution.
- Combining multiple sensor modalities further enhances decoding accuracy, with each added modality contributing measurable gains in $\textrm{R}^{2}$ scores across participants.

These results underscore the importance of both sensor placement and multimodal sensor fusion for reliable myoelectric decoding, while also highlighting the influence of participant-related factors on hand motion decoding. The evaluation included data from 35 participants with a balanced distribution of female and male participants and a wide age range. While this is sufficient to reliably detect major effects on decoding performance, it may not be adequate to detect smaller effects or to fully disentangle interacting participant-related factors. Thus, larger datasets are necessary for future investigations. In future work, we aim to investigate the impact of sensor modalities and muscle regions on task-specific decoding performance, which will help further optimize sensor configurations for practical applications and could enable the design of more effective myoelectric systems.

## Data Availability

The MyoKi database analyzed within this work has been previously published and is available in the figshare repository (https://figshare.com/s/353a15d58e5d25db2359).
